# PERSEUS-IT 24-month analysis: a prospective observational study to assess the effectiveness of intravitreal aflibercept in routine clinical practice in Italy in patients with neovascular age-related macular degeneration

**DOI:** 10.1007/s00417-022-05679-6

**Published:** 2022-05-05

**Authors:** Massimo Nicolò, Francesco Ciucci, Marco Nardi, Barbara Parolini, Andrea Russo, Andrea Scupola, Salvatore Torregrossa, Maria Vadalà, on behalf of the PERSEUS-IT study investigators

**Affiliations:** 1 Clinica Oculistica - DiNOGMI, University of Genoa, Ospedale Policlinico San Martino IRCCS, Genoa, Italy; 2 San Pietro Fatebenefratelli Hospital, Rome, Italy; 3 University of Pisa, Pisa, Italy; 4 Clinica Sant’Anna, Brescia, Italy; 5 University of Catania, Catania, Italy; 6 Fondazione Policlinico Universitario “A. Gemelli” IRCCS, Rome, Italy; 7 Villa Sofia Cervello Hospital, Palermo, Italy; 8 BIND Department, University of Palermo, Palermo, Italy

**Keywords:** Aflibercept, Intravitreal injections, Neovascular age-related macular degeneration, Observational study, Treatment outcomes

## Abstract

**Purpose:**

PERSEUS-IT (NCT02289924) was a prospective, observational, 2-year study evaluating the effectiveness and treatment patterns of intravitreal aflibercept (IVT-AFL) in patients with neovascular age-related macular degeneration (nAMD) in routine clinical practice in Italy.

**Methods:**

Treatment-naïve patients with nAMD receiving IVT-AFL per routine clinical practice were enrolled. The primary endpoint was mean change in visual acuity (VA; decimals) from baseline to month (M) 12 and M24. Outcomes were evaluated for the overall study population and independently for the 2 treatment cohorts: regular (3 initial monthly doses, ≥ 7 injections by M12, and ≥ 4 injections between M12 and M24) and irregular (any other pattern).

**Results:**

Of 813 patients enrolled, 709 were included in the full analysis set (FAS); VA assessments were available for 342 patients at M12 (FAS1Y, 140 regular and 202 irregular) and 233 patients at M24 (FAS2Y, 37 regular and 196 irregular). In the overall FAS, the mean ± SD change in VA from baseline to M12 and M24 was + 0.09 ± 0.24 and + 0.02 ± 0.25 decimals, and there was a statistically significant difference between the regular and irregular cohorts in both FAS1Y (*p* = 0.0034) and FAS2Y (*p* = 0.0222). Ocular treatment-emergent adverse events were reported in 4.1% (*n* = 33/810 [safety set]) of patients.

**Conclusion:**

In PERSEUS-IT, clinically relevant functional and anatomic improvements were observed within the first 12 months of IVT-AFL treatment in routine clinical practice in Italy in patients with treatment-naïve nAMD. These gains were generally maintained across the 2-year study. The safety profile of IVT-AFL was consistent with prior studies.

**Trial registration number:**

ClinicalTrials.gov Identifier: NCT02289924.

**Date of registration:**

November 13, 2014. 
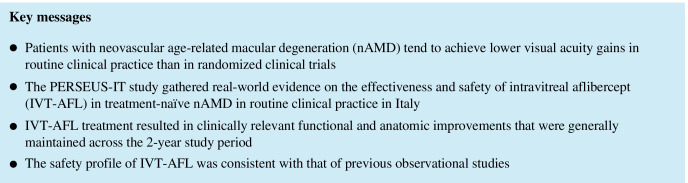

**Supplementary Information:**

The online version contains supplementary material available at 10.1007/s00417-022-05679-6.

## Introduction

Anti-vascular endothelial growth factor (anti-VEGF) therapies, such as intravitreal aflibercept (IVT-AFL), ranibizumab, and brolucizumab, have demonstrated robust improvements in visual outcomes in patients with neovascular age-related macular degeneration (nAMD) in randomized controlled trials [1–6]. However, real-world evidence (RWE) from observational studies shows that patients tend to receive fewer anti-VEGF injections in routine clinical practice, particularly after the first year of treatment, and achieve lower visual acuity gains than typically observed in clinical trials [7, 8].

Previous observational studies on the effectiveness of IVT-AFL in the treatment of nAMD include the 2-year prospective PERSEUS study of treatment-naïve and previously treated patients in Germany [9, 10] and the 4-year retrospective and prospective RAINBOW study in treatment-naïve patients in France [11–13].

In the German PERSEUS study, 28.0% and 6.5% of patients had received regular treatment by the end of year 1 and year 2, respectively (defined as IVT-AFL injections every 8 weeks [q8w] after 3 initial monthly injections, with ≥ 7 injections in year 1 and ≥ 4 injections in year 2); 37.9% of the original 803 patient cohort were still under observation by the end of year 2 [10]. In the RAINBOW study, 19.8% and 13.6% of patients had received regular treatment by the end of year 1 and year 2, respectively (defined as IVT-AFL injections q8w after 3 initial monthly injections, with ≥ 6 injections in year 1, and injections at the discretion of the treating physician in year 2) [12]. In both studies, visual acuity gains were greater in patients who received regular IVT-AFL injections than in those who received irregular treatment.

In Italy, the only currently available RWE was generated in small, retrospective studies [14, 15]. The 2-year PERSEUS-IT study aimed to generate much-needed RWE on the real-world effectiveness and treatment patterns of IVT-AFL among treatment-naïve patients with nAMD in routine clinical practice in Italy.

## Methods

### Study design

PERSEUS-IT (NCT02289924) was a 2-year, prospective, multicenter, observational study to assess the real-world effectiveness and treatment patterns of IVT-AFL in treatment-naïve patients with nAMD. The study enrolled 813 patients and was conducted across 37 university hospitals and eye clinics in Italy between January 2015 and March 2019. All treatment decisions, including the decision to treat with IVT-AFL, were made by the attending physician, according to their local practice, and IVT-AFL was prescribed in accordance with the terms of the marketing authorization.

The study was conducted in accordance with the Declaration of Helsinki, and the guidelines of the European Medicines Agency and the International Council for Harmonization Guideline E3: Good Clinical Practice. The protocol and any amendments were reviewed and approved by each study site’s Independent Ethics Committee or Institutional Review Board before and during the study. All patients provided written informed consent.

### Patients and procedures

The key inclusion criteria were treatment-naïve patients with nAMD who received IVT-AFL in accordance with the local Summary of Product Characteristics (SmPC). Exclusion criteria were any prior or concomitant therapy with another therapeutic agent for nAMD, as well as the contraindications listed in the local SmPC, which included scars, fibrosis, or atrophy involving the center of the fovea.

Patients were retrospectively grouped into regular and irregular treatment cohorts using the approach described in the German PERSEUS study [10]. The classification criteria for these cohorts were prespecified in the statistical analysis plan and were based on injection numbers and the interval between injections. Treatment was considered regular during the first 12 months if initiated with 3 monthly (− 1 week/ + 2 weeks) doses of 2 mg IVT-AFL and ≥ 7 injections were received overall; all other treatment patterns were considered irregular. Treatment was considered regular during the entire 24 months if initiated with 3 monthly doses of 2 mg IVT-AFL, ≥ 7 injections were received during the first year, and ≥ 4 injections were received during the second year; all other treatment patterns were considered irregular. As PERSEUS-IT was an observational study, there were no prespecified retreatment criteria; treatment decisions were made at the discretion of the attending physician, provided that these followed the local SmPC. The initial visit, follow-up visits, and end of observation visit all took place according to routine clinical practice.

Visual acuity was assessed in this real-world study per routine clinical practice at each study center using decimal values (the most frequently used scale in Italy). Online Resource 1 provides a conversion table for decimals, logMAR values, and letter scores for reference.

Either time-domain or spectral-domain optical coherence tomography (OCT) could be used to measure the central retinal thickness (CRT). When the CRT assessment was performed using time-domain OCT, the CRT measurement was adjusted by adding 43.1 µm to fit the measurement of spectral-domain OCT as previously described [16]. If the OCT instrument was not recorded or unknown, the measurement was considered to be performed with spectral-domain OCT and no adjustment was performed. These OCT assessments, as well as fundoscopy and fluorescein angiography, were conducted at the discretion of the attending physician.

### Study endpoints

The primary endpoint was mean change in visual acuity (VA; decimals) from baseline to 12 and 24 months (both − 29/ + 30 days). The secondary endpoints included the mean number of injections within 4, 6, 12, and 24 months; the mean time between injections; the mean time between visits; the mean number of clinical visits (injections only), monitoring visits (no injections), and combined visits (visits for injections and monitoring); the mean change in CRT from baseline to 12 and 24 months; the time between the first nAMD symptoms and diagnosis; and the time from diagnosis to the start of treatment. Data from only one eye (the study eye) per patient were used to evaluate the primary and secondary endpoints. The study eye was defined as the eye for which IVT-AFL treatment was initiated; where both eyes were treated, the study eye was the eye with the worst visual acuity at the start of therapy or diagnosis.

Safety was assessed throughout the study period, and ocular adverse events (AE) were reported for both the study eye and fellow eye. All AEs were summarized using the Medical Dictionary for Regulatory Activities coding system. AEs were considered to be treatment-emergent if they started after the first IVT-AFL injection or, at most, 30 days after the last injection. Safety analyses were performed on treatment-emergent adverse events (TEAEs); events that were not treatment-emergent were listed without further analysis.

### Statistical analysis

To estimate the change in VA from baseline to the end of the study, a standard deviation (SD) of 16 ETDRS letters was assumed (based on the SDs observed in the VIEW studies [1, 17]), which required a sample size of 667 patients. Therefore, an enrollment target of at least 800 patients was established, assuming that the data from 17% of patients would not be usable for analysis. This provided a two-sided 95% confidence interval (CI) for a single mean that extended 1.2 letters from the observed mean with a 95% CI of 2.4 letters, and a CI based on the large sample *z* statistic (calculated using nQuery Advisor v.5.0; Statistical Solutions Ltd., Cork, Ireland).

The safety analysis set (SAS) included all patients who received ≥ 1 IVT-AFL injection. The overall full analysis set (FAS) included all patients in the SAS with a VA assessment in the study eye at baseline and ≥ 1 post-baseline VA assessment ≥ 5 days after the previous injection. The year 1 FAS (FAS1Y) included all patients in the FAS who had a VA assessment between 330 and 390 days after the first injection of IVT-AFL and were assessed ≥ 5 days after the previous injection. The year 2 FAS (FAS2Y) included all patients in the FAS who had a VA assessment between 690 and 750 days after the first injection of IVT-AFL and were assessed ≥ 5 days after the previous injection.

VA and CRT outcomes were evaluated at baseline, month 4, and every 2 months until month 24 for the overall study population and independently for the regular/irregular treatment cohorts. To explore these outcomes, the analysis included the patients’ last measurements observed within − 29/ + 30 days of each time point (where more than 1 measurement occurred in the same time window, the measurement closest to the respective time point was considered for analysis).

The statistical analysis was exploratory and contained analytical and descriptive segments. Continuous variables were described by sample statistics (i.e., mean, standard deviation, minimum, median, quantiles, and maximum) and categorical variables were described using frequency tables displaying absolute and relative frequencies. The study did not aim to confirm or reject predefined hypotheses; however, an exploratory *t*-test for independent samples was performed to test differences in VA between regularly and irregularly treated patients, as well as CRT changes from baseline to 12 and 24 months. To further evaluate differences in visual acuity between regularly and irregularly treated patients, a linear mixed model for repeated measures was implemented to evaluate the cohort, time point, and cohort by time point interactions for all time points within 12 months and 24 months. An unstructured correlation matrix was considered in the model and missing data were assumed to be missing at random. Statistical analyses were performed with the Statistical Analysis System v9.4 software (SAS Institute Inc., Cary, NC, USA).

## Results

### Patients

The study enrolled 813 patients, of whom 810 received ≥ 1 IVT-AFL injection and comprised the SAS (Online Resource 2). A total of 471 of the patients who enrolled (57.9%) completed the study, defined as a study duration longer than 21 months; 772 patients (95.0%) completed 4 months, 737 (90.7%) completed 6 months, 646 (79.5%) completed 12 months, and 307 (37.8%) completed 24 months. Of the 327 patients who did not complete the study, 182 (55.7%) were lost to follow-up and 48 patients (14.7%) discontinued treatment. Of those who discontinued treatment, 9 withdrew following the patient’s decision and 39 discontinued for “other reasons” (including 10 for lack of efficacy).

The overall FAS comprised 709 patients (87.2% of enrolled patients). The FAS1Y group consisted of 342 patients (regular cohort, 140 patients; irregular cohort, 202 patients) and the FAS2Y group consisted of 233 patients (regular cohort, 37 patients; irregular cohort, 196 patients).

The baseline demographics and disease characteristics are listed in Online Resource 3 for the overall FAS and in Online Resource 4 for FAS1Y and FAS2Y according to the regularly and irregularly treated cohorts. In the overall FAS, the mean ± SD age of the patients was 77.7 ± 7.3 years (range: 52–97 years) and 60.1% were female (Online Resource 3). The mean baseline VA was 0.34 ± 0.24 decimals, and the mean baseline CRT was 382 ± 125 µm. There were no marked differences in the baseline VA and OCT findings between the FAS1Y and FAS2Y cohorts or between the regularly and irregularly treated cohorts (Online Resource 4). Some minor differences were observed between the fluorescein angiography results of regularly and irregularly treated patients, particularly for FAS2Y, in which more irregularly treated patients had predominantly classic choroidal neovascularization (CNV; 52.5% vs 35.7% in regularly treated patients) and more regularly treated patients had non-classic occult CNV (50.0% vs 29.5% in irregularly treated patients). The time between symptom onset and diagnosis of nAMD was ≤ 2 weeks in 30.0% of patients (*n* = 212), between 2 weeks and 1 month in another 30.0% of patients (*n* = 212), and > 1 month in 40.0% of patients (*n* = 283). Following diagnosis, the mean time to IVT-AFL treatment was 34 ± 112 days.

### Treatment exposure

Over the first 12 months in the overall FAS, patients had a mean ± SD of 3.7 ± 1.9 visits for injections only, 3.1 ± 2.1 monitoring visits (without injections), and 0.9 ± 1.7 combined visits for both monitoring and injections. Between months 12 and 24, there was a mean of 2.4 ± 1.9 injection-only visits, 4.2 ± 2.4 monitoring visits, and 0.8 ± 1.6 combined visits. Thus, over the 2-year study period, there was a mean of 5.9 ± 3.2 injection-only visits, 7.0 ± 4.0 monitoring visits, and 1.7 ± 3.0 combined visits. The mean time between injections in the overall FAS was 2.4 ± 1.1 months, whereas the mean time between visits (any visit type) was 1.7 ± 0.6 months.

Patients completing 12-month follow-up (FAS1Y) received a mean of 5.6 IVT-AFL injections over 12 months, whereas patients completing 24-month follow-up (FAS2Y) received 3.1 injections between months 12 and 24, and 8.6 injections in total over the full 2-year study period (Table 1). In the FAS1Y cohort, regularly and irregularly treated patients received a mean of 7.3 and 5.0 injections within the first 12 months, respectively. In the FAS2Y cohort, regularly and irregularly treated patients received a mean of 7.6 and 5.7 injections in the first 12 months, 5.5 and 2.5 injections between months 12 and 24, and 12.2 and 7.7 injections in total over the full 2-year study period, respectively. The distribution of the number of injections over the study period is shown in Online Resource 5.

**Table 1:** Mean number of IVT-AFL injections over 12 and 24 months of treatment

		0–12 months	13–24 months	0–24 months
Overall FAS (*N* = 709)		5.6 ± 1.6	3.1 ± 1.8	8.6 ± 2.6
FAS1Y	Regular (*n* = 140)	7.3 ± 0.5	–	–
	Irregular (*n* = 202)	5.0 ± 1.3	–	–
FAS2Y	Regular (*n* = 37)	7.6 ± 0.6	5.5 ± 1.0	12.2 ± 1.0
	Irregular (*n* = 196)	5.7 ± 1.5	2.5 ± 1.6	7.7 ± 2.3

Values are mean ± SD. *FAS* full analysis set; *FAS1Y* year 1 FAS; *FAS2Y* year 2 FAS; *IVT-AFL* intravitreal aflibercept; *SD* standard deviation

### Functional outcomes

In the overall FAS, at months 12 and 24, the mean ± SD change in VA from baseline was + 0.09 ± 0.24 and + 0.02 ± 0.25 decimals, respectively (Online Resource 6). In the 572 patients (80.7%) who received 3 initial monthly doses of IVT-AFL, the mean VA increased from 0.35 ± 0.24 decimals at baseline to 0.41 ± 0.27 at month 4 and 0.42 ± 0.28 at month 6. In the 137 patients (19.3%) who did not receive 3 initial monthly injections, the mean VA increased from 0.32 ± 0.22 (baseline) to 0.37 ± 0.26 (month 4) and 0.37 ± 0.30 decimals (month 6). By month 12, there was little difference between the 2 arms (the mean change in VA from baseline to month 12 was 0.09 ± 0.25 and 0.08 ± 0.22 decimals for patients with and without the 3 initial monthly doses, respectively; note that only descriptive statistics were generated for these data).

**Fig. 1:**
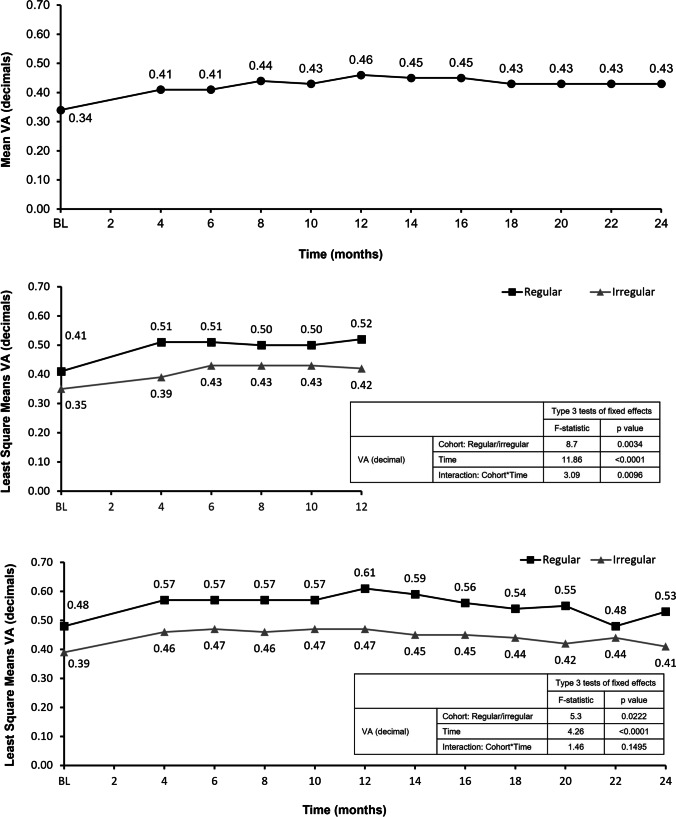
Trend in VA from baseline. **a** Absolute mean VA for the overall FAS over 24 months, **b** least square mean VA from baseline to month 12 for the FAS1Y by regular and irregular cohort, and **c** least square mean VA from baseline to month 24 for the FAS2Y by regular and irregular cohort. BL, baseline; FAS, full analysis set; FAS1Y, year 1 FAS; FAS2Y, year 2 FAS; VA, visual acuity

In the FAS1Y, the mean ± SD VA at baseline was 0.38 ± 0.25 decimals in the total population, and 0.41 ± 0.25 and 0.35 ± 0.24 decimals in the regular and irregular cohorts, respectively. The mean change in VA from baseline to month 12 was + 0.09 ± 0.24 decimals (overall), + 0.11 ± 0.24 decimals (regular cohort), and + 0.07 ± 0.24 decimals (irregular cohort). The mean difference in the change in VA from baseline to month 12 between the regular and irregular cohorts was 0.04 decimals, which was not statistically significant (95% CI: − 0.01, 0.10; *p* = 0.0963). To further evaluate differences in visual acuity between regularly and irregularly treated patients, a repeated-measures model was used. Across year 1, the difference in trend between the regular and irregular cohorts was statistically significant (*p* = 0.0034), with VA higher at each time point in the regularly treated cohort; the time effect (*p* < 0.0001) and interaction between time and cohort (*p* = 0.0096) were also statistically significant (Fig. 1b).

In the FAS2Y, the mean ± SD VA at baseline was 0.41 ± 0.26 decimals in the total population, and 0.48 ± 0.28 and 0.39 ± 0.25 decimals in the regular and irregular cohorts, respectively. The mean change in VA from baseline to month 24 was + 0.02 ± 0.25 decimals (overall), + 0.05 ± 0.22 decimals (regular cohort), and + 0.02 ± 0.25 decimals (irregular cohort). The mean difference in the change in VA from baseline to month 24 between the regular and irregular cohorts was 0.03 decimals, which was not statistically significant (95% CI: − 0.06, 0.11; *p* = 0.5458). In a repeated-measures model, the difference in trend between the regular and irregular cohorts across year 2 was statistically significant (*p* = 0.0222), with VA higher at each time point in the regularly treated cohort; the time effect was also statistically significant (*p* < 0.0001), whereas the interaction between time and cohort was not (*p* = 0.1495) (Fig. 1c).

### Anatomic outcomes

**Fig. 2:**
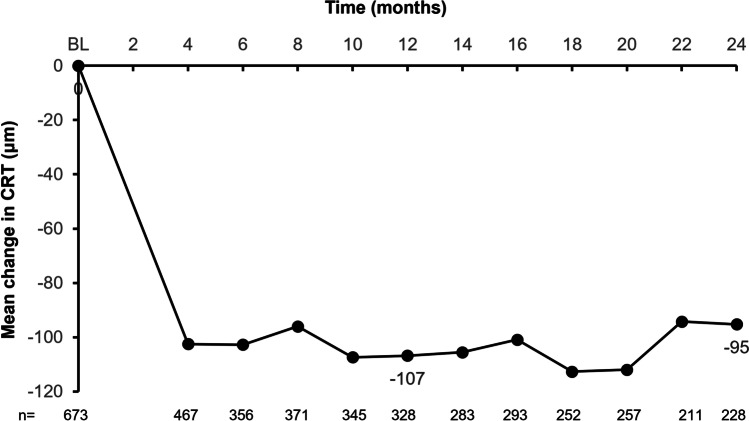
Mean change in CRT from baseline to month 24 for the overall FAS. CRT was not recorded for all patients for whom optical coherence tomography was performed. BL, baseline; CRT, central retinal thickness; FAS, full analysis set

At months 12 and 24 in the overall FAS, the mean ± SD change in CRT from a baseline of 382 µm was − 107 ± 120 µm (reduced to 276 µm) and − 95 ± 118 µm (reduced to 274 µm), respectively (Fig. 2).

In the FAS1Y, the mean CRT at baseline was 383 ± 121 µm overall, with a baseline of 380 ± 113 µm and 384 ± 127 µm in the regular and irregular cohorts, respectively. The mean change from baseline to month 12 was − 108 ± 120 µm overall; − 102 ± 109 µm for the regular cohort, and − 113 ± 129 µm for the irregular cohort. The mean difference of 12 µm (95% CI: − 16, 39) in CRT change from baseline to month 12 between regularly and irregularly treated patients was not statistically significant (*p* = 0.4067).

In the FAS2Y, the mean CRT at baseline was 369 ± 118 µm overall, with a baseline of 379 ± 122 and 368 ± 118 µm in the regular and irregular cohorts, respectively. The mean change from baseline to month 24 was − 94 ± 117 overall, − 85 ± 114 for the regular cohort and − 96 ± 118 for the irregular cohort. The mean differences in CRT change from baseline between the regular and irregular cohorts at month 12 (2 µm [95% CI: − 42, 46; *p* = 0.9283]) and month 24 (11 µm [95% CI: − 32, 55; *p* = 0.6061]) were not statistically significant. The OCT assessments for most patients (*n* = 643) were performed using spectral-domain OCT; time-domain OCT was used for 11 of the remaining patients, and the instrument used was unknown for 36 patients.

### Safety

Of the 813 enrolled patients, all but 3 (*n* = 810, 99.6%) received ≥ 1 injection of IVT-AFL and were included in the SAS, and patients included in the SAS were treated for a mean duration of 13.7 ± 8.0 months. Overall, 9.0% (*n* = 73) of patients experienced TEAEs (Table 2). Ocular TEAEs were reported in 4.1% (*n* = 33) of patients, with the most common TEAE being cataracts (0.6%; *n* = 5). No cases of endophthalmitis, intraocular inflammation, or retinal vasculitis were reported. Five patients (0.6%) reported 6 serious ocular TEAEs in the study eye (*n* = 1 each for macular hole, retinal detachment, retinal hemorrhage, and vitreous hemorrhage; *n* = 2 for retinal pigment epithelial tear); although none of these events were judged by the attending clinical investigator to be related to the study drug, 3 of the events (retinal detachment, *n* = 1; retinal pigment epithelial tear, *n* = 2) were considered related to the intravitreal injection procedure, and 2 of the affected patients permanently discontinued IVT-AFL. Systemic TEAEs were reported in 44 patients (5.3%) with the most common occurring in 3 patients (femur fracture [0.4%]). Three drug-related TEAEs were reported: 2 ocular TEAEs (macular fibrosis and lack of response to treatment) and 1 non-ocular TEAE (acute myocardial infarction considered to be serious). Fifteen deaths (1.9%) were reported during the 2-year study, and none were considered to be related to IVT-AFL treatment.

**Table 2:** Safety overview

Number of patients (%)	Safety analysis set *N* = 810
Any TEAE	73 (9.0)
All ocular TEAEs^a^	33 (4.1)
Cataract	5 (0.6)
Retinal pigment epithelial tears	4 (0.5)
Vitreous hemorrhage	3 (0.4)
Fibrosis	3 (0.4)
Serious ocular TEAEs^b^	5 (0.6)
Retinal pigment epithelial tear	2 (0.3)
Macular hole	1 (0.1)
Retinal detachment	1 (0.1)
Retinal hemorrhage	1 (0.1)
Vitreous hemorrhage	1 (0.1)
Treatment-related ocular TEAEs	2 (0.3)
Discontinuation due to ocular TEAEs	11 (1.4)
Non-ocular TEAEs	44 (5.4)
Serious non-ocular TEAEs	34 (4.2)
Treatment-related non-ocular TEAEs^c^	1 (0.1)
Discontinuation due to non-ocular TEAEs	7 (0.9)
Deaths^b^	15 (1.9)

^a^Only ocular TEAEs occurring in more than 2 patients are reported here. ^b^None of the serious ocular TEAEs or deaths were considered to be IVT-AFL-related. ^c^Acute myocardial infarction, considered serious. *IVT-AFL* intravitreal aflibercept; *TEAE* treatment-emergent adverse event

## Discussion

PERSEUS-IT was the first prospective, multicenter, observational study to evaluate the use of IVT-AFL in routine clinical practice in Italy. Treatment-naïve patients with nAMD achieved improvements in functional and anatomic outcomes with IVT-AFL treatment that were evident within the first 12 months and generally maintained across the 24-month study period. Based on a repeated-measures model, the difference between the least square mean VA was significantly different between the patients in the regular and irregular IVT-AFL treatment cohorts. This outcome is consistent with the 1-year and 2-year findings of the PERSEUS Germany [9, 10] and RAINBOW studies [11, 12], in which patients who received regular treatment achieved greater visual acuity gains than those who did not.

In the German PERSEUS study, treatment-naïve patients who received regular treatment achieved a mean visual acuity gain of + 8.0 letters (baseline: 52.8) compared with + 4.0 letters (baseline 53.7) among those who received irregular treatment in year 1 [9], and the trends observed after 2 years of treatment were similar to those observed in year 1 (+ 6.3 letters in the regular cohort and + 3.3 letters in the irregular cohort [free LOCF populations]) [10]. In year 1 of RAINBOW, treatment-naïve patients who received regular treatment achieved a mean visual acuity gain of + 7.6 letters (baseline: 58.4), compared with + 5.5 letters among those who received irregular treatment but had 3 initial monthly injections (baseline: 56.5) [12]. In year 2, a mean visual acuity gain of + 4.9 and + 4.0 letters was achieved with regular and irregular treatment with the initial monthly injections, respectively; for patients who received irregular treatment without the 3 initial monthly injections, visual acuity increased by 0.1 letters in the first year of treatment and decreased by 2.6 letters by the end of year 2 (baseline: 56.0). Similarly, a retrospective study by the UK Aflibercept Users Group (based on electronic medical records from 1083 patients over 2 years) found that more regular IVT-AFL treatment was associated with superior visual acuity outcomes in nAMD [18].

The visual acuity gains achieved within the first 12 months of IVT-AFL treatment in PERSEUS-IT were similar to, although slightly lower than, those reported in PERSEUS Germany and RAINBOW (acknowledging, however, that the difference in visual acuity measurement units in PERSEUS-IT makes comparisons more complex). Although PERSEUS-IT showed long-term maintenance of visual acuity gains up to month 24, there was a decline in the VA change from baseline observed over the last visits, particularly for the irregular cohort, which suggests that more frequent monitoring and injections may have been required toward the end of year 2. A similar trend in declining VA over the last visits was observed in AURA, an observational study of ranibizumab [8].

The safety profile of IVT-AFL over the 2-year study was consistent with that of prior observational studies [9–13] and no unexpected safety signals were identified. The incidence of retinal pigment epithelial tear during anti-VEGF therapy in PERSEUS-IT is similar to that reported for untreated cases of nAMD; there is no clear evidence of differing risk according to anti-VEGF agent use or type [19].

Administering long-term treatment in real-world settings can present significant hurdles in achieving optimal outcomes for patients with nAMD [7]. Treatment regimens are standardized based on the results of randomized controlled trials [20]; however, in Italy, for example, these standards are not always applied in clinical practice [21]. In a recent retrospective case series of 439 patients with treatment-naïve nAMD in Italy, there was no significant change in VA from baseline at 1 or 2 years following treatment with IVT-AFL or ranibizumab in a pro re nata regimen in routine clinical practice over 2 years [14]; the authors noted that the reasons for this lack of improvement might have included less strict monitoring of patients and elongated injection intervals compared with clinical trials. Furthermore, a population-based study in Italy focusing on data from 2010 to 2016 reported that only 28% of patients prescribed IVT-AFL during this period received 3 initial monthly doses according to the local SmPC, and wide heterogeneity was observed in the patients’ treatment intervals [21]. However, in PERSEUS-IT, in which visual gains were observed and maintained, the proportion of patients receiving regular IVT-AFL injections in the first year of treatment (41%) was relatively high. This may be attributable to increasing physician awareness of the suboptimal outcomes associated with under-treatment. By comparison, 20–28% of patients received regular treatment in the German PERSEUS and RAINBOW studies [9–12].

High treatment discontinuation rates are one of the known limitations in the evaluation of observational studies [7]. Approximately 41% of the patients enrolled in PERSEUS-IT discontinued the study, and 67% were excluded from the FAS2Y analysis because they did not have appropriate measurements within the required timeframes. This is consistent with other observational studies in nAMD (e.g., 45% of enrolled patients completed the AURA study of ranibizumab at 2 years [8] and only 35% of patients were included in the German PERSEUS FAS2Y [10]). This relative under-treatment of patients, along with delays between the diagnosis and treatment of nAMD, can be associated with progressive vision loss in routine clinical practice [22, 23]. A better understanding of the risk factors associated with non-adherence and non-persistence with treatment is needed to improve patient care and outcomes in the management of nAMD [7].

A further limitation of PERSEUS-IT in terms of comparing the results to previous studies is that all descriptive statistics for continuous variables related to visual acuity were reported and evaluated in decimal values, which are a reduction of the Snellen fraction, instead of a letter score or logMAR [24]. Different units of measure complicate comparisons, and although conversions between different units can be performed, such conversions can be misleading. For example, Snellen charts have an irregular progression in size and are often truncated such that the data may not follow a normal distribution, implying that parametric analyses may be best avoided; this cannot be mitigated by merely converting the data to logMAR or ETDRS letters [24]. It was, therefore, decided to describe the primary outcomes in decimals rather than applying a conversion to different scales.

Given the nature of this observational study, the aim of which was to evaluate real-world effectiveness and treatment patterns, all decisions related to patient treatment were at the discretion of the attending physician, provided that these followed the recommendations of the SmPC in Italy. This has the inherent limitation that different patients might have been exposed to diverse treatment patterns, leading to increased variability in treatment outcomes. Methodological limitations to this study include the use of different OCT machines and modalities (time-domain and spectral-domain OCT) at the various clinical centers, with adjustment of the time-domain OCT data being required prior to analysis.

The strengths of the PERSEUS-IT study include the prospective design, the large number of enrolled patients, the 24-month study duration, and the high mean age of the participants (78 years) in line with what would be expected for this real-world population of patients with nAMD. Furthermore, PERSEUS-IT was conducted in 37 centers across Italy, providing a variety of real-world clinical settings from which to generate data.

## Conclusions

In PERSEUS-IT, treatment with IVT-AFL resulted in clinically relevant functional and anatomic improvements in treatment-naïve patients with nAMD in routine clinical practice in Italy. Functional and anatomic improvements were evident within the first 12 months and were generally maintained across the 24-month study period. Although a decline in mean VA was observed over the last visits of year 2, the general trend observed in the visual acuity gains in PERSEUS-IT highlights the importance of ongoing appropriate treatment in routine clinical practice to maintain and optimize patients’ vision outcomes. Consistent with other studies, treatment-naïve patients who received 3 initial monthly IVT-AFL injections followed by regular treatment over the study period experienced improved functional outcomes over 24 months compared with patients who received irregular treatment. The safety profile of IVT-AFL was consistent with that of previous studies.

### Electronic supplementary material

Below is the link to the electronic supplementary material.

Supplementary file1 (PDF 222 KB)

## Data Availability

Availability of the data underlying this publication will be determined according to Bayer’s commitment to the EFPIA/PhRMA “Principles for responsible clinical trial data sharing.” This pertains to scope, time point, and process of data access. As such, Bayer commits to sharing upon request from qualified scientific and medical researchers’ patient-level clinical trial data, study-level clinical trial data, and protocols from clinical trials in patients for medicines and indications approved in the United States (US) and European Union (EU) as necessary for conducting legitimate research. This applies to data on new medicines and indications that have been approved by the EU and US regulatory agencies on or after January 1, 2014. Interested researchers can use www.clinicalstudydatarequest.com to request access to anonymized patient-level data and supporting documents from clinical studies to conduct further research that can help advance medical science or improve patient care. Information on the Bayer criteria for listing studies and other relevant information is provided in the study sponsors section of the portal. Data access will be granted to anonymized patient-level data, protocols, and clinical study reports after approval by an independent scientific review panel. Bayer is not involved in the decisions made by the independent review panel. Bayer will take all necessary measures to ensure that patient privacy is safeguarded.

## Appendix

PERSEUS-IT study sites and investigators

**Table:**

Study site	Investigator
Azienda Ospedaliera Cardinale Giovanni Panico, Tricase	Antonio Basurto
Azienda Ospedaliera Nazionale SS Antonio e Biagio e Cesare Arrigo, Alessandria	Andrea Coggiola
Azienda Ospedaliera Ospedali Riuniti Villa Sofia − Cervello, Palermo	Salvatore Torregrossa Antonino Pioppo
Azienda Ospedaliera San Camillo Forlanini, Roma	Francesco Contento Alessandro Carboni Raffaele Santoro
Azienda Ospedaliera Spedali Civili, Brescia	Francesco Semeraro Elena Gambicorti Giorgio Fusardi
Azienda Ospedaliera Universitaria Policlinico Paolo Giaccone, Palermo	Maria Vadalà Salvatore Cillino
Azienda Ospedaliera Universitaria Policlinico Umberto I, Roma	Rosalia Giustolisi
Azienda Ospedaliera, Desenzano Del Garda	Vincenzo Pucci
Azienda Ospedaliero − Universitaria “Policlinico-Vittorio Emanuele”, Catania	Vincenza Bonfiglio
Azienda Ospedaliero − Universitaria di Ferrara, Arcispedale Sant’Anna, Ferrara	Paolo Perri
Azienda Ospedaliero Universitaria Careggi, Firenze	Gianni Virgili
Casa di Cura Beato Palazzolo, Bergamo	Cristiano Luiselli
Clinica Oculistica Universitaria di Pisa, Pisa	Marco Nardi Chiara Posarelli
Fondazione Policlinico A Gemelli Università Cattolica Del Sacro Cuore, Roma	Andrea Scupola
Istituto Clinico Sant’Anna, Brescia	Barbara Parolini Gianluca Besozzi Sajish Pinackatt Roberta Panzani
Istituto Ospedaliero, Cremona	Pierfilippo Sabella
Ospedale Bartolomeo Eustacchio, San Severino Marche	Vincenzo Ramovecchi
Ospedale Civile, Carrara	Franco Passani
Ospedale Civile, Legnano	Giuseppe Trabucchi Ermengarda Marziani
Ospedale Fatebenefratelli e Oftalmico, Milano	Stefano Erba
Ospedale Maggiore, Parma	Monica Camparini Sally Louise Williams Alessandra Pareti
Ospedale Policlinico San Martino, Clinica Oculistica, Genova	Massimo Nicolò
Ospedale Policlinico San Martino, Uo Oculistica, Genova	Tommaso Rossi Daniele Ferrari
Ospedale Policlinico San Matteo, Pavia	Giulio Vandelli
Ospedale Policlinico SS Annunziata, Chieti	Leonardo Mastropasqua Lisa Toto
Ospedale San Giuseppe, Milano	Giuseppe Moretti
Ospedale San Pietro Fatebenefratelli, Roma	Francesco Ciucci Cristiano De Gaetano
Ospedale Sant’Anna di San Fermo della Battaglia, Como	Massimo Conti
Ospedale Santa Maria Alle Scotte, Siena	Claudio Traversi Fiorella Fusco
Ospedale Santa Maria Degli Angeli, Pordenone	Antonio Manfrè
Ospedale Santa Maria Degli Angeli, Putignano, Bari	Antonio Acquaviva
Ospedale SS Trinità, Borgomanero	Vito Belloli Caterina Pisano
Ospedale Valduce, Como	Giampiera Gambaro
Policlinico di Modena, Modena	Gianmaria Cavallini
Presidio Ospedaliero 1, “San Donato”, Arezzo	Andrea Romani
Presidio Ospedaliero C Sperino, Oftalmico, Torino	Claudio Panico
Presidio Ospedaliero di Savona, Cairo Montenotte Ospedale San Paolo, Savona	Gianmaria Venturino Cristina Siniscalchi
