# Supplementary material for: PERSEUS-IT 24-month analysis: a prospective observational study to assess the effectiveness of intravitreal aflibercept in routine clinical practice in Italy in patients with neovascular age-related macular degeneration
Source: Graefes Arch Clin Exp Ophthalmol. 2022 May 5;260(10):3185–95. doi: 10.1007/s00417-022-05679-6 (PMC9477902; doi:10.1007/s00417-022-05679-6)
Supplement: Supplementary file 1 — Supplementary file1 (PDF 222 KB) [file 417_2022_5679_MOESM1_ESM.pdf]

Supplementary Information

PERSEUS-IT 24-month analysis: a prospective observational study to assess the effectiveness of intravitreal aflibercept in routine clinical practice in Italy in patients with neovascular age-related macular degeneration

Massimo Nicolò[1]*, Francesco Ciucci[2], Marco Nardi[3], Barbara Parolini[4], Andrea Russo[5], Andrea Scupola[6], Salvatore Torregrossa[7], Maria Vadalà[8], on behalf of the PERSEUS-IT investigators

[1]Clinica Oculista – DiNOGMI, University of Genoa, Ospedale Policlinico San Martino IRCCS, Genoa, Italy
[2]San Pietro Fatebenefratelli Hospital, Rome, Italy
[3]University of Pisa, Pisa, Italy
[4]Clinica Sant'Anna, Brescia, Italy
[5]University of Catania, Catania, Italy
[6]Fondazione Policlinico Universitario "A. Gemelli" IRCCS, Rome, Italy
[7]Villa Sofia Cervello Hospital, Palermo, Italy
[8]BIND Department, University of Palermo, Palermo, Italy

***Corresponding author:**
Massimo Nicolò
E-mail: massimonicolo@gmail.com
ORCID iD: 0000-0002-7824-3091

Online Resource 1. Conversions between decimals, logMAR values, and letter scores

Decimal	LogMAR	Letter score
2.0	−0.3	100
1.6	−0.2	95
1.25	−0.1	90
1.0	0	85
0.8	0.1	80
0.63	0.2	75
0.5	0.3	70
0.4	0.4	65
0.32	0.5	60
0.25	0.6	55
0.2	0.7	50
0.16	0.8	45
0.125	0.9	40
0.1	1.0	35
0.08	1.1	30
0.063	1.2	25
0.05	1.3	20
0.04	1.4	15
0.03	1.5	10
0.025	1.6	5
0.02	1.7	–
0.016	1.8	–
0.0125	1.9	–
0.01	2.0	–

Table adapted from Elliot DB (2016) Ophthalmic Physiol Opt 36(4):355-358. https://doi.org/10.1111/opo.12310.
MAR, minimum angle of resolution.

Online Resource 2. Patient disposition

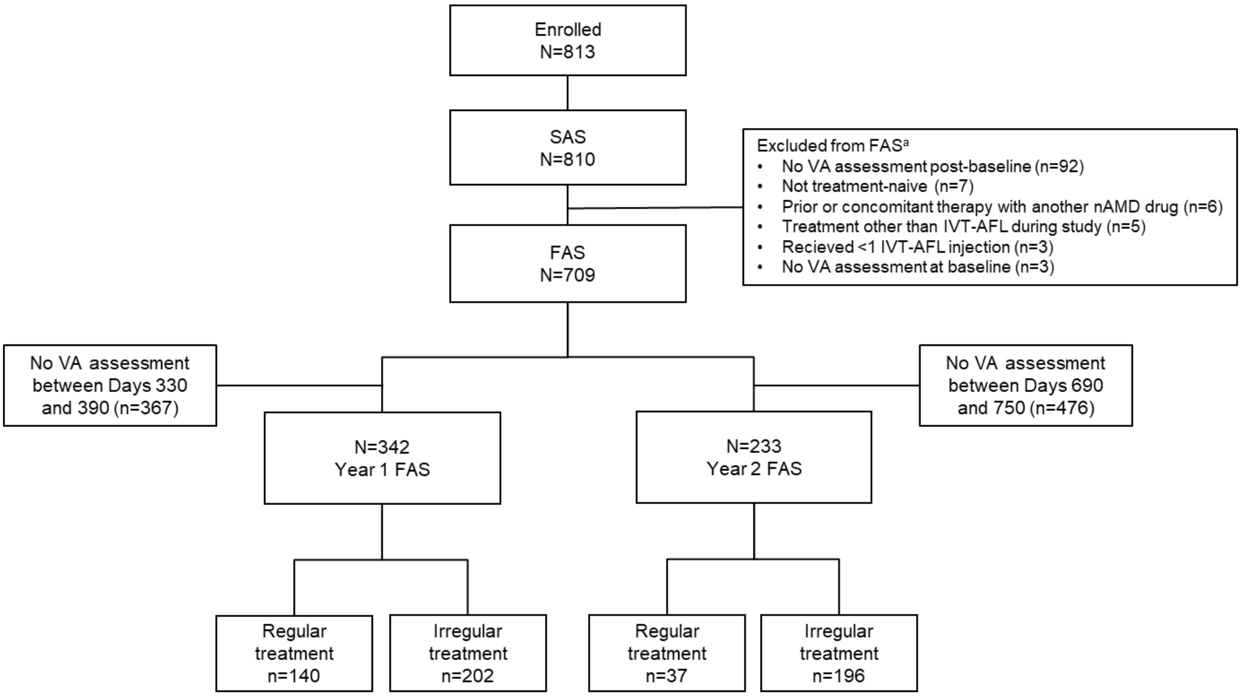

[a]Patients may have had ≥1 reason for exclusion. FAS, full analysis set; IVT-AFL, intravitreal aflibercept; nAMD, neovascular age-related macular degeneration; SAS, safety analysis set; VA, visual acuity.

Online Resource 3. Patient baseline demographics and disease characteristics in the FAS

	**FAS (N=709)**
**Age, years**	77.7±7.3
**Female, n (%)**	426 (60.1)
**Mean time from diagnosis to IVT-AFL treatment, days**	34±112
**VA in the study eye, decimals**	0.34±0.24
**CRT, μm[a]**	382±125
**CNV type by FA, n (%)[b]**	
Predominantly classic	243 (55.5)
Minimally classic	62 (14.2)
Occult, with no classic	110 (25.1)
No CNV or CNV not active	11 (2.5)
Unknown	12 (2.7)
**Presence of retinal fluid, n (%)[c]**	
Subretinal fluid	525 (76.2)
Intraretinal fluid	504 (73.1)

Values are mean±SD unless otherwise stated. [a]n=673; [b]Fluorescein angiography was conducted in 438 patients. [c]Optical coherence tomography was conducted in 689 patients. CNV, choroidal neovascularization; CRT, central retinal thickness; FA, fluorescein angiography; FAS, full analysis set; IQR, interquartile range; SD, standard deviation; VA, visual acuity.

Online Resource 4. Patient baseline demographics and disease characteristics in the FAS1Y and FAS2Y

	FAS1Y (N=342)		FAS2Y (N=233)	
	**Regular (n=140)**	**Irregular (n=202)**	**Regular (n=37)**	**Irregular (n=196)**
**VA in the study eye, decimals**	0.41±0.25	0.35±0.24	0.48±0.28	0.39±0.25
**CRT, μm[a]**	380±113	384±127	379±122	368±118
**CNV type by FA, n (%)[b]**				
Predominantly classic	58 (60.4)	69 (51.9)	10 (35.7)	64 (52.5)
Minimally classic	8 (8.3)	22 (16.5)	2 (7.1)	16 (13.1)
Occult, with no classic	26 (27.1)	34 (25.6)	14 (50.0)	36 (29.5)
No CNV or CNV not active	2 (2.1)	4 (3.0)	1 (3.6)	1 (0.8)
Unknown	2 (2.1)	4 (3.0)	1 (3.6)	5 (4.1)
**Presence of retinal fluid, n (%)[c]**				
Subretinal fluid	106 (76.8)	152 (78.0)	30 (83.3)	147 (76.2)
Intraretinal fluid	97 (70.3)	142 (72.8)	20 (55.6)	136 (70.5)

Values are mean±SD unless otherwise stated. Proportions (%) are calculated relative to the number of patients assessed in each cohort. [a]CRT was assessed in 325 patients in FAS1Y and 220 patients in FAS2Y; [b]FA was conducted in 229 patients in FAS1Y and 150 patients in FAS2Y. [c]Fluid status was assessed by OCT in 333 patients in FAS1Y and 229 patients in FAS2Y. CNV, choroidal neovascularization; CRT, central retinal thickness; FA, fluorescein angiography; FAS, full analysis set; FAS1Y, all patients in the FAS with a VA assessment at Month 12; FAS2Y, all patients in the FAS with a VA assessment at Month 24; OCT, optical coherence tomography; SD, standard deviation; VA, visual acuity.

Online Resource 5. Distribution of the number of injections over (**a**) the first 12 months in 607 patients completing 12 months' follow-up, (**b**) between Month 12 and Month 24 in 298 patients completing 24 months' follow-up, and (**c**) the 24-month study period, also in 298 patients completing 24 months' follow-up.

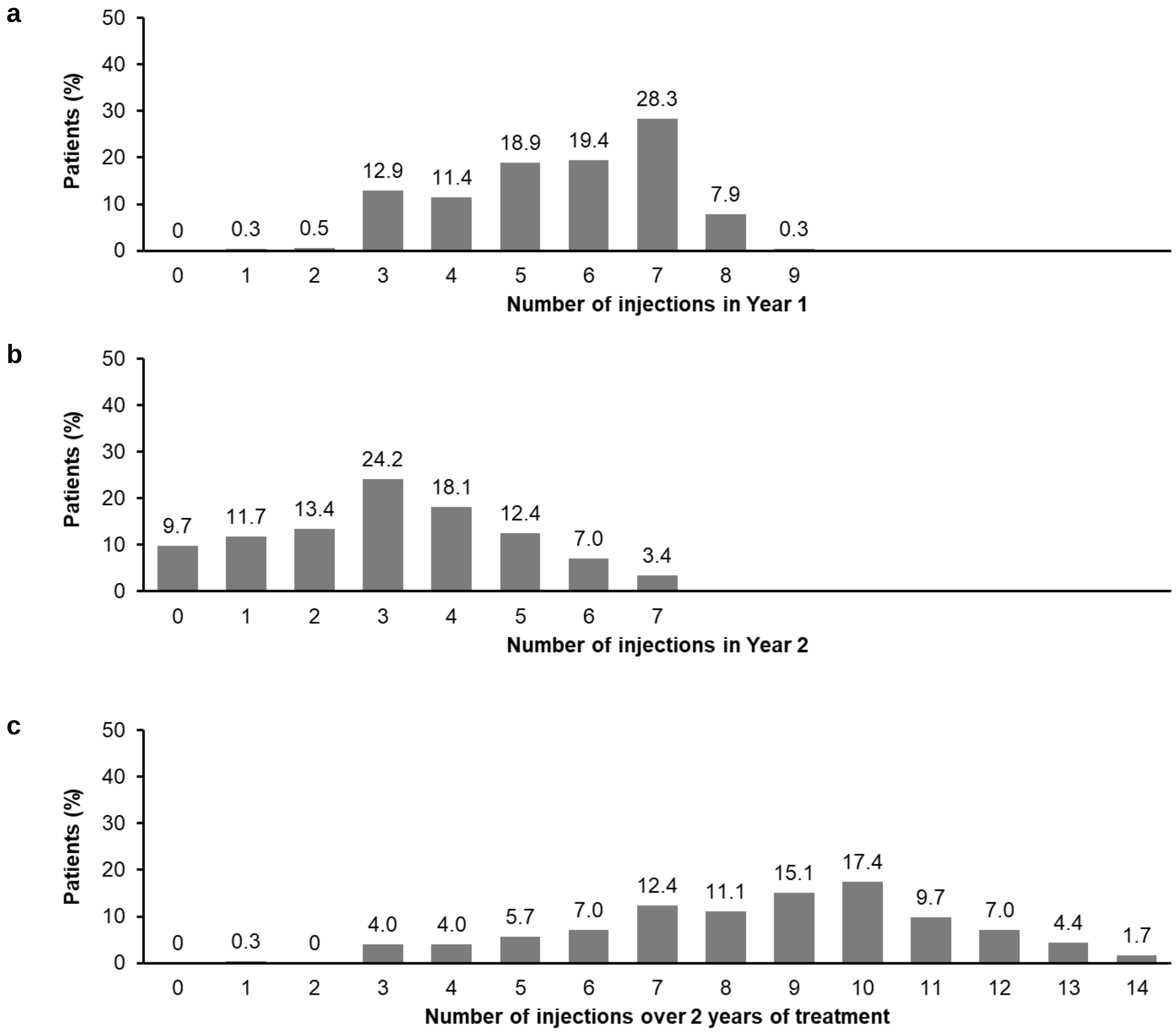

Online Resource 6. Mean change in visual acuity (VA) over time in the overall full analysis set. The VA observed within a time window of −29/+30 days from each of the indicated time points was included. Where >1 VA assessment occurred within the same time window, the assessment closest to the time point was used.

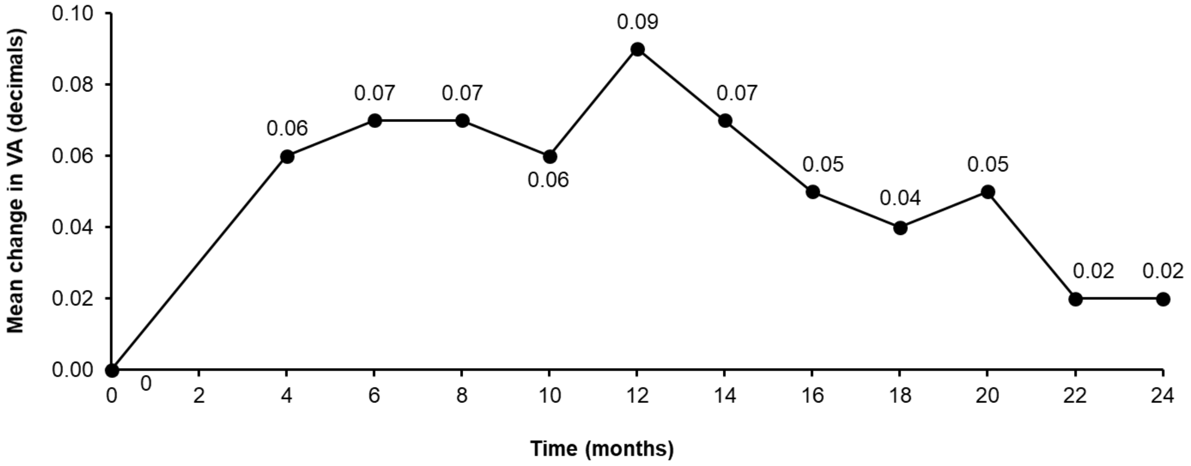

.
